# A Hyperacute Presentation of Small Cell, Non-Nodal Mantle Cell Lymphoma

**DOI:** 10.1155/crh/9912698

**Published:** 2025-07-18

**Authors:** Jodi Chiu, Mark Crowther

**Affiliations:** ^1^Department of Medicine, Division of Hematology, Western University Schulich School of Medicine and Dentistry, London, Canada; ^2^Department of Medicine, Division of Hematology, McMaster University, Hamilton, Canada

**Keywords:** leukostasis, lymphoreduction, mantle cell lymphoma, tumour lysis syndrome

## Abstract

Mantle cell lymphoma (MCL) is an aggressive mature B-cell non-Hodgkin lymphoma. Patients often present with lymphadenopathy, early satiety, and B-symptoms. Presentation with hyperleukocytosis is rare. The small cell, non-nodal variant of MCL tends to be less aggressive, have lower mitotic rates, and mimics morphology of chronic lymphocytic leukemia (CLL). We present a 79-year-old woman admitted to hospital with generalized weakness, gait instability, and dyspnea; she was found to have a white count of 550 × 10^9^/L, hemoglobin of 30 g/L, and platelets of 49 × 10^9^/L. She had biochemical evidence of poor tissue perfusion. Peripheral blood smear demonstrated lymphocytosis with smudge cells. After aggressive red blood cell transfusion, she was managed as leukostasis with concurrent tumour lysis syndrome (TLS). She was administered intravenous fluids, rasburicase, allopurinol, and escalating doses of prednisone for lymphoreduction. Her mentation and biochemical evidence of shock improved. Although we initially had high suspicion for CLL, her flow cytometry raised concerns for MCL. Cytogenetics confirmed *t* (11; 14) rearrangement. This case is the first to discuss a severe, aggressive presentation of a small variant, leukemic non-nodal MCL. We also review the role of steroids in leukostasis and concurrent warm autoimmune hemolytic anemia, in a centre where leukapheresis is unavailable.

## 1. Introduction

Mantle cell lymphoma (MCL) is an uncommon, aggressive mature B-cell non-Hodgkin lymphoma (NHL), comprising around 5%–7% of all lymphomas [1]. MCL occurs more frequently in men [1]. Patients often present with lymphadenopathy, splenomegaly, B-symptoms, and bone marrow involvement at the time of diagnosis. On a molecular level, it is classically defined by *t*(11; 14)(q13:q32) translocation resulting in the overexpression of cyclin D1 gene [2]. MCL often confers a poor prognosis, associated with early relapse and inferior survival.

Studies have noted that 77%–92% of patients with MCL have peripheral blood involvement by flow cytometry; however, most cases do not have overt leukemic involvement at the time of diagnosis [3, 4]. The leukemic phase of MCL is defined as absolute monoclonal lymphocytosis > 10 × 10^9^/L [5]. Presentation of hyperleukocytosis secondary to MCL is exceedingly rare, with only a handful of case reports in the literature. Leukemic MCL results in circulating lymphoma cells that vary in morphology depending on the underlying MCL subtype.

The World Health Organization (WHO) recognizes four morphological variants of MCL, including classic, small cell, blastoid, and pleomorphic variants. Blastoid and pleomorphic variants are classically more aggressive, while the small cell variant typically has lower mitotic rates and mimics the morphology of chronic lymphocytic leukemia (CLL)/small lymphocytic lymphoma (SLL) [6]. More recently, the WHO has identified the leukemic non-nodal MCL (nnMCL) as a distinct subtype of MCL, characterized by leukemic expression, splenomegaly, and no or minimal nodal involvement. This is unlike classical MCL, which typically presents with widespread lymph node involvement. Previously, leukemic involvement by MCL was thought to confer poor prognosis; however, more recent literature has described the leukemic nnMCL subtype, particularly the small cell variant, to have a more indolent course [2, 7, 8]. We report a case of MCL, small cell variant, presenting with hyperleukocytosis (white blood count [WBC] > 500 × 10^9^/L), with neurological and cardiopulmonary leukostasis.

## 2. Case Presentation

A 79-year-old female with a past medical history of well-controlled schizophrenia and hypertension presented to the Emergency Department with a three-week history of generalized weakness, frequent falls, gait instability, and shortness of breath. She did not endorse chest pain, focal neurological deficits, constitutional symptoms, or early satiety.

Physical examination was significant for a temperature of 36.4°C, pulse of 105 beats per minute, blood pressure of 100/46, and oxygen saturation of 99% on 2L nasal prong. She appeared to have yellow ‘sallow' skin with pale conjunctiva but otherwise did not appear in distress. No palpable cervical or axillary lymphadenopathy or hepatosplenomegaly were appreciated. There was no evidence of leukemia cutis, petechiae, or significant ecchymosis.

Significant laboratory findings on presentation are summarized in Table 1.

Her bloodwork 2 years prior demonstrated normal leukocyte and lymphocyte counts, and a creatinine of 52 μmol/L.

Chest x-ray demonstrated ill-defined airspace disease in the right upper, mid, and lower lung zones with mild perihilar interstitial prominence. CT head was negative for acute intracranial process. ECG demonstrated ST depressions diffusely, concerning for myocardial ischemia. A presumptive diagnosis of CLL with rapid doubling time was made.

She was admitted to the medical step-down unit, with the Hematology consult service following closely. She was emergently transfused four units of packed red blood cells and initiated on maintenance intravenous (IV) hydration at 100 mL per hour for management of both her leukostasis and TLS. We administered IV rasburicase 7.5 mg once, followed by allopurinol 300 mg orally followed by 150 mg thrice per day.

Several hours after initiation of these medications, we administered IV methylprednisolone 25 mg once. As the patient tolerated the dose with no significant laboratory evidence of tumour lysis syndrome (TLS), we administered a higher dose of IV methylprednisolone 100 mg on the second day, again with good response. On the third day, we escalated the dose of IV methylprednisolone to 150 mg, with another dose of rasburicase 5 mg. On the fourth day, we maintained her dose at 150 mg intravenously per day, while awaiting return of the various definitive laboratory tests that had been sent for pathological analysis.

Her WBC trend with lymphocyte and neutrophil differentials from Days 1–5, and Day 9, along with administered methylprednisolone doses at each point are summarized in Table 2.

Clinically, she had resolution of her dyspnea and confusion. After initial management and stabilization over the course of 4 days, the patient was transferred to the Hematology Oncology unit in another tertiary hospital within the city for further management.

Peripheral blood flow cytometry revealed a monoclonal B cell population representing 90% of total leukocyte count, dimCD19+, CD20+, CD38-, dim/partial CD5+, CD10, heterogeneous CD79+, CD23-, CD103-, and CD200-, with kappa restriction. Bone marrow and lymph node flow cytometry yielded similar immunophenotyping, with bone marrow aspirate consistent with extensive marrow infiltration by lymphoproliferative neoplasm. Cytogenetics from her bone marrow confirmed IGH/CCND1 rearrangement. SOX11 was negative, which is more frequently associated with leukemic nnMCL. CT chest, abdomen, pelvis was positive for splenomegaly, with spleen measuring 15.9 cm in coronal plane. As such, her results confirmed our concerns for leukemic MCL, small cell variant, with extensive marrow infiltration, resulting in hyperleukocytosis.

After bone marrow aspirate and biopsy, the patient was initiated on bendamustine/rituximab, 9 days after initial presentation. At this point, her white count was 145.7 × 10^9^/L, and had continued to decrease post-transfer with no further steroid administration. Postchemotherapy administration, the patient became pancytopenic. Unfortunately, the patient had multiple complications, including transfusion-associated circulatory overload, candida bacteremia, and delirium. Her goals of care were changed to comfort, and she passed away 1 month after presentation.

## 3. Discussion

We report a case of MCL, small lymphocyte variant, presenting with an aggressive clinical course of hyperleukocytosis and leukostasis. To our knowledge, this is the second case report on leukemic MCL with a circulating small lymphocyte phenotype resulting in leukostasis [9]. There have been a small number of other case reports of peripheralizing MCL of the prolymphocytoid, pleomorphic, and blastoid variants, causing hyperleukocytosis and leukostasis [1, 10–13]. Pleomorphic and blastoid variants are classically more aggressive, especially in comparison to small cell MCL, and, as such, are more likely to present with leukemic transformation [2, 8].

Hyperleukocytosis is a laboratory abnormality, commonly defined by a total WBC > 100 × 10^9^/L [14]. Leukostasis is symptomatic hyperleukocytosis, where the aggregation of WBCs results in poor tissue perfusion, and subsequent tissue hypoxia [14]. It is diagnosed clinically, in any patient with hyperleukocytosis, and concurrent cardiopulmonary and/or neurological signs and symptoms.

Leukostasis is more commonly associated with acute leukemia [14]. It is significantly less common amongst chronic leukemias and mature lymphoproliferative neoplasms, unless WBC exceeds > 500 × 10^9^/L, as these mature cells are smaller, and have lower metabolic and mitotic rates [14].

The current patient presented with hyperleukocytosis of 543.3 × 10^9^/L, manifesting as gait instability, confusion, and dyspnea. Chest x-ray demonstrated pulmonary infiltrates. She had biochemical evidence of tissue hypoxia with hyperlactatemia and metabolic acidosis, as well as electrocardiographic and laboratory evidence of myocardial ischemia. While our patient's presentation and evidence of tissue hypoxia could have also been explained by profound anemia, we could not rule out leukostasis in the presence of marked hyperleukocytosis.

Along with IV fluid resuscitation, we used methylprednisolone for its lymphoreductive properties. Despite the risk of increasing blood viscosity, we could not avoid transfusion given our patient's profound anemia. We initiated low dose steroids before dose escalation to monitor her clinical and laboratory response. Specifically, we were concerned for worsening TLS. Although hydroxyurea is a common cytoreductive therapy in leukapheresis, we were concerned of its myelosuppressive effects, and potential worsening of the patient's bicytopenia. We were also suspicious for secondary warm autoimmune hemolytic anemia (AIHA) as a contribution to her significant anemia. Steroids are often the first line treatment for warm AIHA. As such, we were hopeful that steroid administration would have a two-pronged effect in both lymphoreduction, and hemolysis suppression.

Our patient's WBC and lymphocyte differential, albeit with marked clinical improvements, were fluctuant during her 4-day course of steroids. We attributed her paradoxical increase in leukocyte quantitation in part to varying hemoconcentration, and demargination of neutrophils secondary to the high doses of steroids. Her WBC was ultimately steroid responsive and continued to decrease from 543.3 on presentation to 149.7 on Day 9 after a 4-day course of steroids, with no further intervention until chemotherapy initiation on Day 9.

Leukapheresis in mature B-cell lymphoproliferative disorders has not been well-studied due to the rarity of leukostasis in this patient population. There have been case reports of peripheralizing MCL patients responding well to leukapheresis, although the majority of these cases have been amongst MCL patients with blastoid, pleomorphic, and prolymphoid variants, which have white cell morphology more representative of acute leukemia [10, 11]. Kwan et al. reports a case of MCL with circulating small lymphocyte phenotype, WBC 465 × 10^9^/L, presenting with severe dyspnea and hypoxemia. Their patient had resolution of respiratory symptoms after one session of leukapheresis [9].

The hospital our patient was initially admitted under did not have leukapheresis capabilities. However, despite lack of access to leukapheresis, our patient demonstrated improvement in her neurological function and hypoxemia after steroid administration. Furthermore, she had resolution in biochemical evidence of tissue hypoxia, as well as electrocardiographic and laboratory markers of myocardial ischemia. Unfortunately, despite prompt initiation of chemotherapy upon transfer, she passed away within 1 month of presentation from multiple malignant and chemotherapy-associated complications.

The outcome of this case highlights the aggressive nature of our patient's underlying lymphoma, and poor prognosis that leukostasis in MCL confers. Most patients presenting with leukostasis secondary to leukemic MCL discussed in case reports die within 6 months of presentation.

In summary, we present a case of small lymphocyte variant MCL that highlights an unusual presentation. Although recent literature have demonstrated that nnMCL, small variant, may have a more indolent clinical course [7, 8], we are the first to report an unusually severe, and aggressive presentation of small variant, leukemic nnMCL with hyperleukocytosis, TLS, and profound anemia. Our patient's WBC was steroid responsive. Escalating steroid administration with close attention for TLS may be considered in mature B-cell lymphoproliferative disorder patients presenting with marked peripheral lymphocytosis and leukostasis, where leukapheresis is unavailable, especially when concurrent secondary warm AIHA is suspected. One limitation to the use of steroids is the potential adverse effect on the diagnostic accuracy of the initial biopsy.

Our case also underscores the importance of remaining vigilant of nonspecific signs and symptoms of leukostasis, particularly in patients with mature B-cell lymphocytosis. Given our patient's unexpected diagnosis of leukemic MCL, small cell variant, our case emphasizes the importance of maintaining a broad differential of the underlying hematological malignancy, so patients can be rapidly and appropriately managed, especially in the case of aggressive lymphoma.

## Figures and Tables

**Table 1:** Laboratory values on presentation to the emergency department.

Blood test	Result	Normal range
Complete blood count and differential		
WBC (× 10^9^/L)	534.3	4–10
Lymphocytes	449.9	1–4
Monocytes	23	0.2–0.8
Neutrophils	34.7	2–7.5
Hemoglobin (g/L)	30	135–170
Platelets (× 10^9^/L)	49	150–400
Peripheral smear	Marked lymphocytosis with smudge cells. No blasts.	
Metabolic panel		
Potassium (mmol/L)	7	3.5–5
Ionized calcium (mmol/L)	1.13	1.09–1.30
Phosphate (mmol/L)	1.59	0.80–1.33
Creatinine (μmol/L)	111	62–120
LDH (U/L)	565	≤ 214
Urate (μmol/L)	594	143–339
Unconjugated bilirubin (μmol/L)	35	3.4–12
Venous blood gas		
pH	7.09	7.32–7.42
Lactate (mmol/L)	4.1	0.9–2.4
Bicarbonate (mmol/L)	10	22–29
Cardiac markers		
Troponin I (peak) (ng/L)	6356	< 30
Coagulation markers		
INR (s)	1.3	0.8–1.1
APTT (s)	27	25–37
D-dimer (ug/L)	30,933	0–500
Fibrinogen (g/L)	1.6	2–3.9

Abbreviations: APTT, activated partial thromboplastin time; INR, international normalized ratio; WBC, white blood count.

**Table 2:** White blood cell trend from presentation until initiation of chemotherapy, with concurrent methylprednisolone administrated doses.

Day of treatment	WBC (× 10^9^/L)	Lymphocyte count (× 10^9^/L)	Neutrophil count (× 10^9^/L)	IV methylprednisolone dose
Day 1	543.3	481	16.5	25 mg

Day 2	331.3	304	15.8	100 mg

Day 3	278.2	No differential	No differential	150 mg

Day 4	387.4	342.4	23.9	150 mg

Day 5	230	205.2	16.2	No steroids or chemotherapy administered
…				
Day 9	149.7	128.6	8.7	

Abbreviation: WBC, white blood count.

## Data Availability

Data sharing is not applicable to this article as no new data were created or analyzed in this study.

## Supporting Information

“CARE Checklist of Information to include when writing a case report” completed and uploaded under Supporting Information.
